# Feasibility of Self-Programming of the Speech Processor Via Remote Assistant Fitting in Experienced Cochlear Implant Users

**DOI:** 10.1055/s-0044-1789194

**Published:** 2025-01-27

**Authors:** Paola Angelica Samuel-Sierra, Maria Valéria Schimidt Goffi-Gomez, Ana Tereza de Matos Magalhães, Ricardo Ferreira Bento, Robinson Koji Tsuji

**Affiliations:** 1 Department of Otolaryngology, Hospital das Clínicas, Faculty of Medicine, Universidade de São Paulo, São Paulo, SP, Brazil

**Keywords:** cochlear implant, deafness, fitting, remote fitting, neural response telemetry

## Abstract

**Introduction** Adults with cochlear implants (CIs) need periodic programming of their speech processors to take advantage of alternative adjustments. However, this requires patients to attend the CI center in person.

**Objectives** To evaluate the feasibility of speech processor (SP) self-programming with remote assistance in CI users. To establish the characteristics of those who could benefit from self-programming.

**Methods** Adults with at least 1 year of experience with their CI, and whose SP was compatible with the use of the remote assistant fitting (RAF) were selected. Maps were created by the RAF from the neural response telemetry (NRT) results, evaluated in the same session with the audiologist. Patients were given 15-days to adjust to either the routine map or the NRT-based one. In the next session, the minimum and maximum stimulation levels (T- and C-levels) of all the maps were compared.

**Results** No statistical difference was found when comparing the T- and C-levels of the map in use, the map adjusted by RAF, and the NRT-based map created by the RAF and adjusted by the patient.

**Conclusion** Self-programming of the SP was safe and feasible in the studied sample of adults, since T- and C-levels were similar between the behavioral and RAF-adjusted maps. We consider it advisable to use the RAF for patients who have insertion of electrodes and at least one functioning; as well as those who do not have changes in anatomy, nor motor and cognitive conditions that prevent RAF usage.

## Introduction

For cochlear implants (CIs), during the speech processor (SP) programming, several parameters can influence the conversion of sound into an electrical signal. These parameters can be set and chosen by the audiologist, according to the needs of each patient.

One of the most important and fundamental parameters for good sound quality, and the most time-consuming programming step, is the search for the minimum levels of electric stimulation, also known as threshold (T) levels, that generate an audible sensation to the patient, as well as the maximum comfort (C) levels. Determining these levels correctly is important since they generate the electric dynamic range, which establishes the sound input to be coded by the SP.

Several methods can be used to determine T and C-levels, targeting efficiency and optimization of programming and sound quality. 1 2 3 In behavioral methods, generally the CI user is asked to refer to the lowest level capable of detecting 100% of the stimulus (T-level), and the intensity at which the level of stimulation is loud, but comfortable (C-level). In objective methods, measures that do not depend on the patient's response are used, such as the electrically evoked compound action potential (ECAP). It is also possible to combine both methods, that is, basing the programming on objective tests and observing patients' reaction or response, making adjustments as necessary. 4 5 6 7 8 9 During the first programming sessions, the initial focus is achieving audibility. As soon as the user becomes more familiar with electrical hearing, fine-tuning tasks can begin to seek better sound quality. 1 These adjustments are individual and vary according to the needs of each patient.

For adults, T and C-levels become stable with approximately 12 months of use. 10 After this period, it is necessary for the patient to visit the CI center periodically to monitor the device, since stimulation level may be influenced by physiological changes, accommodation to electrical stimulation, 7 11 12 13 14 and duration of hearing loss. 15 At our CI center, approximately six returns are planned in the first year of use, to ensure patients' adaptation and audibility. From the first year of use, adults are followed up annually, or according to their needs. Currently, alternatives to this service have been studied, such as remote programming, but for this study we required patients to have access to a center with an internet connection and the specific programming interface. 16 17 18 19 20 21 22 23 24 25 26 27 28

Some SPs currently available on the market, such as those from the Cochlear Corporation (Sydney, NSW, Australia), have remote assistants that allow volume modification, map selection and a screen for the visualization of the functioning of the SP and troubleshooting. The remote assistants, when enabled by the audiologist, can also change T- and C-levels, perform neural response telemetry (NRT), and even create a new map. With this tool, patients are able to make adjustments to their SP without the need for face-to-face assistance, and create a new map based on their neural responses.

With an increasing number of CI users and expanding indication criteria for CIs, centers are overloaded and have less available time to follow-up on newer patients, who still need SP monitoring and review. 29 30 31 Additionally, throughout this study, we have experienced a crucial situation of social isolation in many countries, which made it impossible for patients to attend periodic monitoring. Therefore, having the possibility of self-programming can enable experienced patients to make adjustments to their own CI by programming it according to their needs, seeking better sound quality in their daily lives without the need of personal audiologist assistance. 32 There are previous reports from other countries, 30 31 33 34 35 but not from any in Latin America. If the fitting with the remote assistant creates map adjustments with stimulation levels that does not lead to discomfort and may even improve patient's satisfaction, the procedure may be considered safe and would reduce the number of appointments to the clinic.

Nevertheless, to use this technology it is important to familiarize ourselves not only with its benefits, but also with its limitations.

The aim of the study was to evaluate the feasibility of self-programming the SP with the remote assistant in adults with CI, identifying whether there are differences between the levels of stimulation generated by the remote control and those programmed maps based on the behavioral methods. Furthermore, to identify the characteristics of those patients who could perform self- programming with their SP.

## Methods

The present is a prospective cross-sectional study approved by Ethics Committee on Research of the Institution (protocol number 1.685.965).

### Inclusion Criteria

- Cochlear CI users, with a receiver stimulator compatible with the AutoNRT Nucleus Freedom 5, CI24RE series, CI422 (Cochlear Corp.), users of SPs, compatible with remote assistants (CR110), such as CP802, Nucleus 5 (CP810).
- Adults (over 18-years-old) with stable maps, an effective use of SP (at least 8 hours a day) for at least 1 year, as reported by the patients, since the CP810 has no datalogging.
- Speech recognition of at least 50% in open set, to guarantee the comprehension of the orders.
- Presence of intraoperative neural response in at least one electrode, as an indicator of possible postoperation NRT response.

### Exclusion Criteria

- Partial insertion of the electrode array.
- Motor or cognitive disabilities that would make it impossible to manipulate the remote assistant.
- Any type of cochlear malformation.

Users who were invited and agreed to participate in the study were introduced to the procedures and signed an informed consent form. There was no compensation for participants who accepted to participate of this study.

Data regarding age, time of CI use, etiology, electrode array model, presence of intraoperative NRT, measured with the Custom Sound EP (Cochlear Corp.) software, and SP model were collected from patient's medical records.

#### Material

**Fig. 1:**
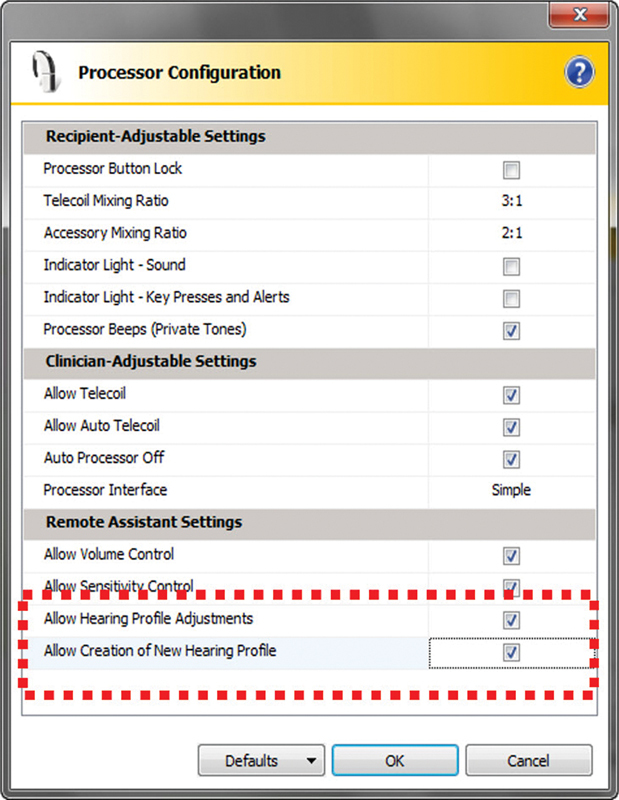
Image of the programming screen with master volume, bass, and treble settings enabled, and a new hearing profile from AutoNRT (highlighted in the dashed line) in the speech processor configuration in the CS 5.2 software (Sidney, Australia).

Remote assistants CR110 with a firmware update achieved using the Custom Sound, v. 4.0 or higher, programming software ( Fig. 1 ), in the configuration of the SP details, fitting adjustments are called Remote Assistant Fitting (RAF) when enabled. 33

**Fig. 2:**
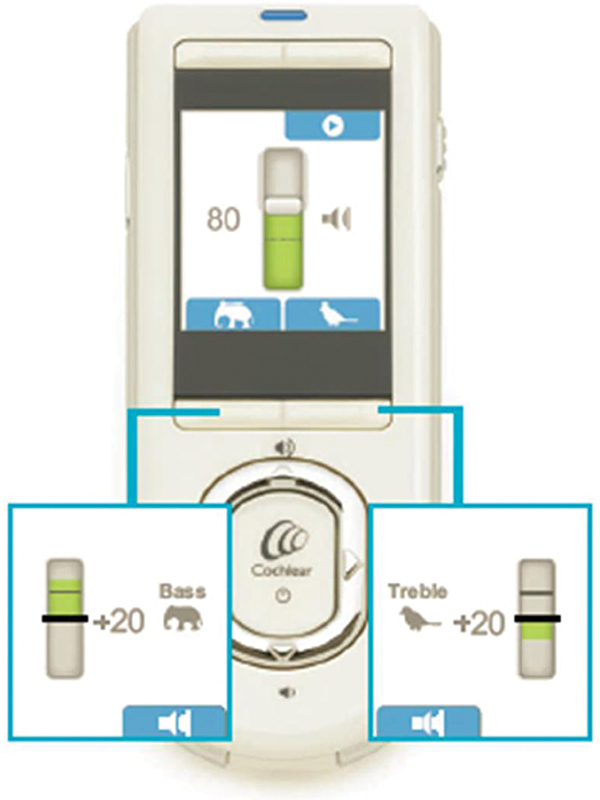
The CR110 remote assistant with the modification functions in master volume (central screen), bass, and treble (smaller frames on the left and right, respectively). 17

The RAF has some main functions, such as: (1) adjusting Master Volume, Bass, and Treble (MVBT) on a map already existing in the SP (created by the audiologist), as shown in Fig. 2 ; (2) measuring automatic neural response telemetry (AutoNRT); 36 and (3) creating a new hearing profile (a map according to the patient's neural response).

The Master Volume refers to a global increase of C-levels on the map in use, with no change in T-levels. When patients can adjust the map created and saved by the audiologist in the SP, the Master Volume function changes only the C-levels. The adjustments are made in increments of 2 current levels (CL, an arbitrary unit in the SP) respecting the map's compliance limit. The CI user can make these changes with the map turned on and the processor microphone activated.

The Bass and Treble are modifications that can be made to emphasize low or high frequencies, respectively. These controls are similar to those suggested by Smoorenburg in 2005. 37 Both are initially set to zero and can only be changed every 2 CL at both ends of the electrode array. They can be modified up or down, with a maximum of 30 current levels, respecting the compliance limits.

**Fig. 3:**
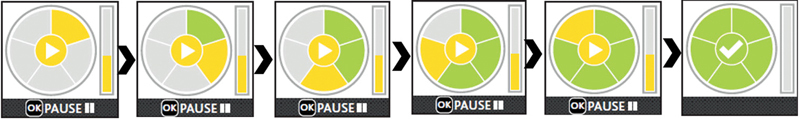
A RAF AutoNRT of five electrodes, where the dashed line represents the threshold survey, and the dark segment represents the threshold already found. Source: Image courtesy of Cochlear Latin America.

Another RAF function is creating a new hearing profile based on the AutoNRT of five electrodes (numbers 22, 16, 11, 6, and 1). To obtain the ECAP thresholds, the system uses the same parameters as the Custom Sound EP, a stimulation rate of 80 Hz (pulses per second), starting the stimulation at 100 CL, increasing the stimulus in 6 CL steps. Upon finding a response, the current level decreases in 3 CL steps until the ECAP threshold is reached. 38 The system stops increasing the level upon either finding a response or reaching the compliance limits or the maximum stimulus level (255). Furthermore, the AutoNRT adjustment duration is around 3 minutes, depending on the thresholds, with higher thresholds taking longer due to more stimulation levels being tested. The ECAP thresholds obtained from the tested electrodes can be used as a basis to establish the stimulation levels that will generate a map. 33 If the RAF AutoNRT does not find a neural response at a chosen electrode, the system automatically switches to an adjacent one. If the user feels discomfort during the measurement, it is possible to interrupt the test or change electrodes ( Fig. 3 ). If no ECAP could be recorded, the RAF system establishes the profile based on default measures from an average population value. 39

At the end of the AutoNRT, the new map is generated, with a 900 Hz stimulation rate and 25 µs pulse width (PW). The map is created with C-levels averaging 50 current units below the thresholds of the neural response. Following the profile scaling method, the C-level profiles are set to be flatter than T ones, since equal loudness contours have been found to be flatter at higher current levels. 39 The average dynamic area per channel is approximately 40 CL. With these levels, the programs are normally inaudible for most CI users, allowing selfadjustments to be made safely.

Subsequently, patients will be able to increase the Master Volume gradually. However, the increase will occur in both T- and C-levels. After reaching comfortable loudness, the Bass and Treble can also ne modified. Ideally, patients should be as comfortable as possible with their hearing levels, being able to state whether sounds are audible and pleasant.

#### Procedures

**Fig. 4:**
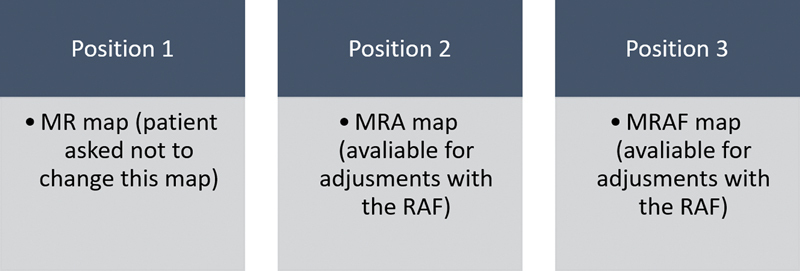
Maps saved in the speech processor after the first visit.

Procedures involved the following stages:

First visit:

a) The map in use by the patient was revised and optimized in order to resolve possible complaints and saved as a Reference Map (MR). Optimization consisted of reviewing the streamlined electrodes 1, 6, 11, 16, and 22, as well as balancing loudness across all the interpolated ones. The map in use was created behaviorally with counted T- and C-levels at comfortable loud stimulus.
b) The MR was saved twice in the SP, in the positions 1 and 2. The patient was asked not to make changes to the map allocated in position 1, to maintain the same parameters adjusted by the audiologist. On the other hand, the map saved in position 2 was enabled for RAF changes according to patients' needs. To make the identification of this map easier in the analysis, it was saved as Reference Map enabled for adjustments (MRA).
c) With the audiologist's assistance, patients used the RAF to perform AutoNRT.
d) From the results of AutoNRT, the RAF created a new hearing profile (new map), according to the scaling method.
e) Still in the presence of the audiologist, patients adjusted the new hearing profile, changing Master Volume, Bass, and Treble functions, in order to create a comfortable map.
f) As practice and support, patients were assisted and guided to learn how these adjustments could be done at home with the RAF, and a new map saved as MRAF Map in position 3.
g) Therefore, at the end of this session, three maps were saved in the SP: in position 1, the MR (map previously used by the patient); in position 2, MRA (map previously used by the patient, available for adjustments in the home experience); and in position 3, the MRAF map (map according to the profile scaling method, available for adjustments in the home experience) ( Fig. 4 ).
h) The function to perform a new AutoNRT and create a new auditory profile was disabled for home experience, but the other adjustment functions were kept enabled.

The patient was instructed to make adjustments according to their preferences in the MRA and MRAF, keeping the MR untouched. The position of the maps was not randomized, so that the patient could have the map in position 1 as a reference, knowing that it was their old map in use, in case there was any discomfort with the adjustments made by the RAF. The use of the MRA and MRAF maps was required for 1 week each, for the listening experience and adaptation with the possibility of adjustments by the RAF during this period.

After 2 weeks of home experience and adjustments with the RAF, patients' subjective opinion about the preferred map was collected, with the question: “After these 2 weeks of experience, among the maps in position 1, 2 or 3, which one have you preferred to use in your daily life?”. Additionally, we applied the virtual analogue scale (VAS) and asked for participants' opinions on the most comfortable map.

Before this study, patients didn't have any kind of experience with the RAF functions, only with the CR110 or CR230 functions (as volume, program, and sensibility of the SP).

#### Statistical Analysis

The electrode array was divided into four groups to analyze the electrical stimulation levels, being apical electrodes (18–22), medial 1 (6–10), medial 2 (11–17), basal (1–5). We divided the 22 electrodes in 4 groups, reflecting the apical, medial, and basal regions, although the medial region had to be further divided, due to the greater number of electrodes. We preferred not to use single electrodes in each region, otherwise we would have disregarded the importance of balancing loudness in the interpolated channels.

Averages of the T- and C-levels of the apical, medial 1, medial 2, and basal electrodes were compared using the Mann-Whitney test among the MR (behavioral), the MRA, and the MRAF.

## Results

A total of 9 users of the CP810 SP (Nucleus 5) participated in this study, all of whom were adults, with a mean age of 46 (22–59) years and an average time of CI use of 39.8 (25–61) months, using the same SP since activation ( Table 1 ). The CI users who participated in this study were familiarized with the use of the remote assistant in their routine for adjusting volume, changing maps, and troubleshooting, but had never used the tools mentioned in this study. For this reason, all of them received guidance on use and handling on the first visit.

**Table 1:** Characterization of participants in relation to sex, age (in years), etiology, electrode array type, and time of CI use (in months)

Sex	N (%)	
** Female**	3 (33.3)	
** Male**	6 (66.6)	
**Age, years**		
** Median (min** – **max)**	44.2 (22–65)	
**Etiology**	**N (%)**	
** Unknown**	4 (44.4)	
** Ototoxicity**	2 (22.2)	
** Meningitis**	1 (11.1)	
** Trauma**	1 (11.1)	
** Genetic**	1 (11.1)	
**Type of electrode array**	**N (%)**	
** Straight**	4 (44.4)	
** Perimodiolar**	5 (55.5)	
**Time of CI use, months**		
** Median (min** – **max)**	49 (19–84)	

**Abbreviation:** CI, cochlear implant.

Of the participants, 6 showed a response in all electrodes tested in AutoNRT, as performed by the RAF ( Table 2 ). Although there were 2 patients with absent responses postoperatively and one with partial presence, all participants allowed the RAF to perform AutoNRT. Some patients reported that the stimulus was too loud, but none of them stopped the test, even those who reached the maximum available current level or the compliance limits.

**Table 2:** Postoperative NRT thresholds, in current levels, in the electrodes 22, 16, 11, 6, and 1 performed by the RAF

Postoperative NRT					
	Electrodes				
	e22	e16	e11	e6	e1
S1	↓	↓	↓	↓	↓
S2	142	151	156	142	171
S3	172	166	169	178	181
S4	157	169	202	172	172
S5	178	193	205	202	190
S6	↓	↓	184	178	172
S7	178	172	166	145	178
S8	↓	↓	↓	↓	↓
S9	229	190	190	178	189

**Abbreviation:** NRT, neural response telemetry; RAF, remote assistant fitting. **Note:** ↓ absent neural response on NRT tested by RAF (reached the maximum current/compliance limit without recording threshold).

Table 3 shows the map parameters of MR and the map created by the RAF from the NRT for each participant. Regarding the frequency allocation table (FAT), both the minimum and maximum frequency remained the same in MR and MRAF, with the minimum registered frequency being 188 and the maximum 7,938 Hz.

**Table 3:** Characteristics of the MR and MRAF, with stimulation rate, maximum, PW and number of active electrodes

	Stimulation rate (pps or Hz)		Maximum		PW (µ)		Active electrodes	
	MR	MRAF	MR	MRAF	MR	MRAF	MR	MRAF
**S1**	900	900	8	8	Var	25	20	22
**S2**	1200	900	8	8	25	25	22	22
**S3**	900	900	10	8	25	25	22	22
**S4**	900	900	8	8	25	25	22	22
**S5**	900	900	12	8	25	25	19	22
**S6**	900	900	12	8	25	25	22	22
**S7**	900	900	12	8	25	25	22	22
**S8**	900	900	8	8	var	25	20	22
**S9**	900	900	12	8	25	25	22	22

**Abbreviations:** MR, reference map; MRAF, reference map created by the remote assistant fitting; pps, pulses per second; Hz, Hertz; PW, pulse width; var: variable pulse width. **Notes:** Variable pulse width was defined as different pulse width values along the electrode array.

**Fig. 5:**
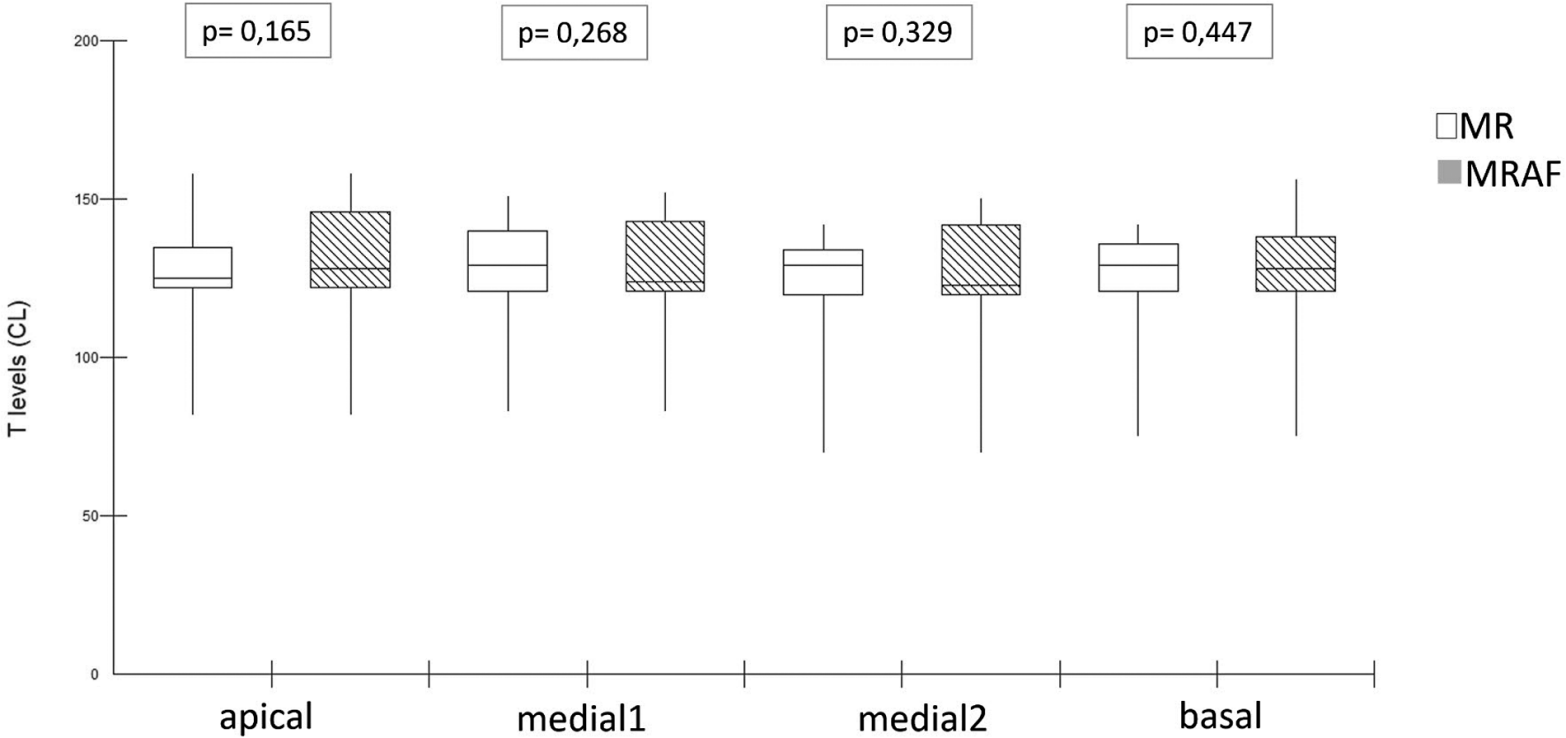
Box plot of average Ts level from the apical, medial, and basal electrodes of the reference map (MR) and the map according to the profile scaling method (MRAF).

**Fig. 6:**
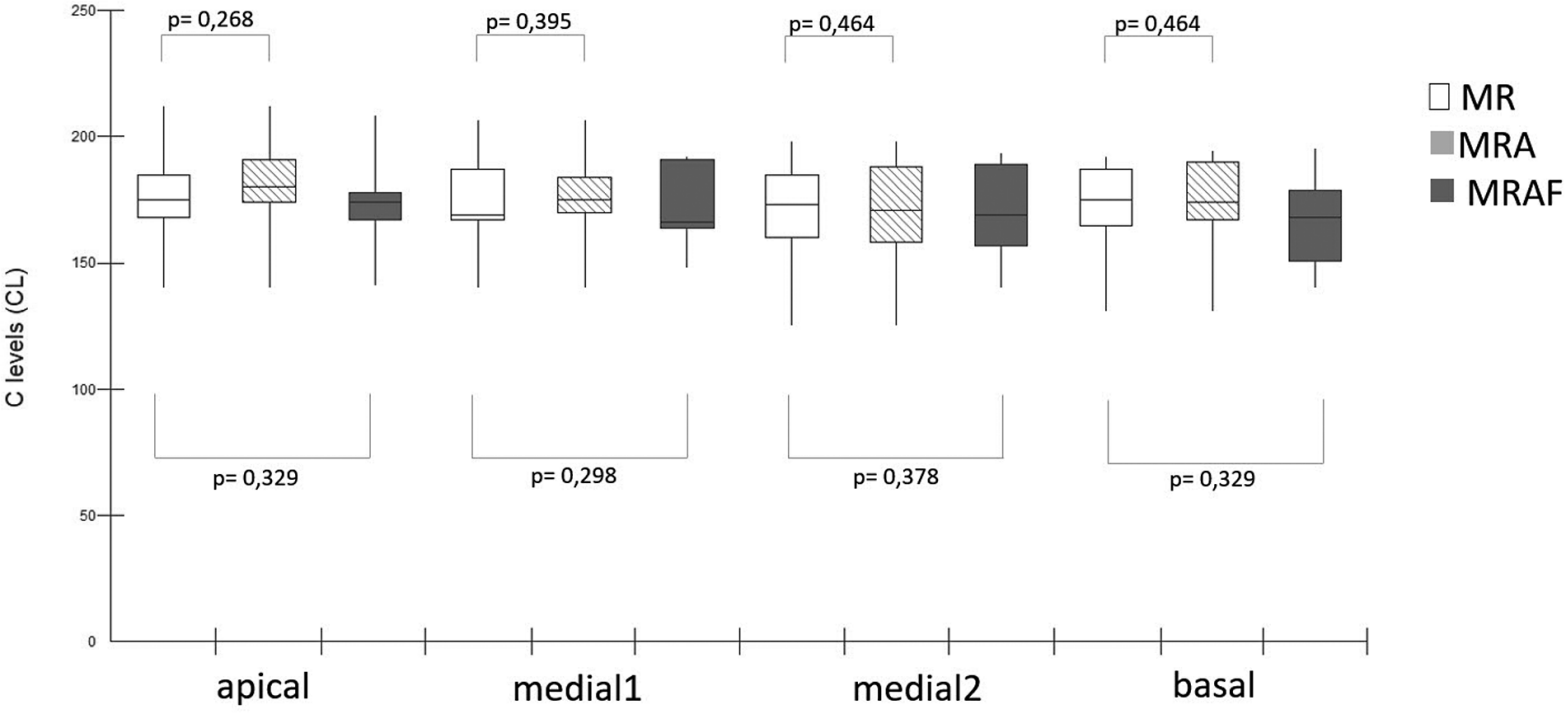
Box plot of average C levels from the apical, medial, and basal electrodes of the reference map (MR), the MR allowed to adjustments (MRA, and the map according the profile scaling method (MRAF).

The box plots ( Figs. 5 and 6 ) show the average of the electrode current units in the MR, MRA, and MRAF. There was no significant difference in C levels between MR and MRA, nor in the T- and C-levels between MR and MRAF. The T-levels between MR and MRA were not compared since the RAF only modifies C-levels on the map in use.

One of the participants (S5) presented nonauditory stimulation with the MRAF. The hearing loss etiology in this case is trauma and, despite a complete insertion, the MR had three electrodes disabled to avoid stimulation of the facial nerve. As these electrodes were activated on the MRAF, the patient again presented facial nerve stimulation. This patient only underwent home experience with the MRA but still presented complaints of discomfort and poor sound quality. Thus, while we recorded the adjustments made by him when trying to improve sound quality, but this participant did not continue the study. Therefore, his preferred map was considered as the one in use (MR).

There was another participant (S2) who had difficulty handling the RAF during the home experience, as it did not allow adjustments to be made due to some technical failure. The participant changed the volume and sensitivity of the microphone, assuming the parameters offered by the RAF were being adjusted. Therefore, it was considered that his preferred map was the MR.

Furthermore, 4 participants asked to keep the self-adjustment function enabled on their SP at the end of the study, as they noticed an improvement in listening comprehension in challenging environments, as well as television and music after adjustments in the RAF.

The preferred maps reported by patients after their home experience point to the MR or MRA, to which they were well accustomed ( Table 4 ).

**Table 4:** Preference of participants in relation to the tested maps

Preferred map	N (%)	
**MR**	4 (44.4)	
**MRA**	4 (44.4)	
**MRAF**	1 (11.1)	

**Abbreviation:** MR, reference map; MRA, reference map enabled for adjustments; MRAF, reference map created by remote assistant fitting and adjusted by the user. **Note:** ↓ absent neural response on NRT tested by RAF (reached the maximum current/compliance limit without recording threshold).

## Discussion

The aim of this study was to evaluate the feasibility of self-programming the SP in adults with the remote assistant, identifying whether there are differences between the stimulation levels generated by the remote control and those on behaviorally programmed maps. We also tried to identify the profile of patients who could benefit from this technology.

The AutoNRT was performed by the RAF on all participants. There were 2 participants who showed no response in all tested electrodes, and one who showed only a partial response (the current reached the maximum limit of the equipment). Although some reported that the stimulus was too loud, none of them requested that the test be stopped. All participants had a neural response during the intraoperative period. As an indication criterion, the presence of intraoperative neural response in at least one electrode was chosen to guarantee a greater chance of neural response in the postoperative period. In the absence of the neural response, the RAF creates a map with levels of stimulation similar to those of patients with neural response, but following the profile based on a preestablished average. 39

Regardless of the parameters preestablished by the audiologist according to the needs of each patient, or of the internal device and the compliance limits needs, we could observe that the RAF creates a map with default parameters (900 Hz per channel of stimulation rate, 8 maximum, PW at 25 µs, and all electrodes activated).

Some patients had maps with variable PW. When the RAF created a new map, the standard was 25 μs. The reduction in PW may cause a decrease in loudness, leading to further increases in the stimulation levels to compensate. The RAF prevents increases when compliance limits are reached. Despite this, one of the participants with variable PW (S1) in the MR, preferred the MRA despite the modifications. For this participant, the T- and C-levels of the preferred map were not only higher but followed a different profile from the map in use, due to bass and treble modifications by the patient.

In our study, we compared the T- and C-levels between the MR, MRA, and MRAF. No significant differences were found, which showed that experienced patients have adjusted based on their auditory preferences, not requiring extreme changes to adapt to the sound. Botros et al. 33 compared conventional programming (performed by the audiologist) with the map made by the RAF from AutoNRT and with the Nucleus Fitting software (simplified programming interface). They found no significant difference in the T- and C-levels among the three maps.

We asked the participants of our study about their preferred map after home experience. Among them, 4 preferred the MRA, 3 chose the MR, 1 preferred the MRAF, and one subject did not complete the study (S5). Vroegop et al. 34 also observed patients' preference for the adjustments made to the map previously in use. We believe that the findings of the present study may have occurred due to stable T- and C-levels and adaptation to the map in use, since 3 participants had the longest use of the CI in the sample (between 71 and 84 months, more than 6 years of CI use). Indeed, Hughes et al. 10 suggest that T- and C-levels in adults stabilize at around 12 months of use.

It is important to consider that the RAF, when used to modify the map made with behavioral measures, only changes C-levels, while it is able to modify T- and C- levels when used to adjust a map created by CR110. However, in both situations, the T-level cannot measure the levels individually.

Among the total of 9 participants, only one (S1) showed preference for the MRAF. In a subjective judgment, the participant referred that this map enabled a greater understanding of speech in open environments, better sound localization, better television sound recognition, and less need for daily volume adjustments. In the analysis of this participant's maps, the MRAF had a reduced dynamic range, with an increase in T- and a reduction in C-levels, mainly in apical and medial electrodes. As mentioned earlier, this participant used a map with variable PW (between 25 and 37µs) and, after having a map created by the RAF, he started to use the same PW on all electrodes.

Participants were asked to adjust the maps as needed during their daily life. The home experience was important for participants to test the adjustments in different environments. Each map was required to be used for 1 week, and participants were instructed on the importance of testing all maps. As experienced users, we believe that they could identify the settings that better suit them.

Even with previous training on RAF use, one participant (S2) had difficulty handling the program during the home experience. The RAF was unconfigured and the participant adjusted volume and sensitivity of the microphone, assuming he was modifying the parameters of Master Volume, Bass, and Treble. Although this was the only case in our group, we believe that the RAF's user interface is not ideal for all patients, since motor and cognitive functions can influence patients' ability to handle and learn. 40

Of the 9 participants, 4 asked to keep the self-adjustment function enabled on their SP at the end of the study period, as they noticed an improvement in listening comprehension in challenging environments, television comprehension, and music after adjustments in the RAF. We theorize that the others have not asked to keep the RAF enabled as it is a new experience. Many users are already adapted to their maps and believe that changes are not necessary, or that it could not benefit their hearing.

For Cullington et al., 35 CI centers offer annual follow-ups, even without the need for interventions. Even in experienced CI users, audiologists can check the SP integrity and detect any deterioration in patients' speech recognition and hearing. For this reason, they believe that remote monitoring could not be offered to all patients but would allow the centers to have greater availability for patients with more complex needs. Govaerts et al. 41 emphasized that the SP's programming demands time from the professional, even in already stable and adapted cases. Therefore, they argued that programming can be a simplified process without losing its effectiveness.

Studies that used the RAF as a tool to enable self-adjustment 30 33 34 35 42 agreed that the possibility can be offered to adults with CI without compromising the efficiency and performance of the SP. However, patients, family members, and professionals must make this decision together, considering each individual's characteristics.

Although the RAF is a tool already available, it was not routinely used in our center before this study. It is important to be aware of the benefits and limitations of this device, in addition to knowing the profile of patients who can and cannot benefit from its use.

The use of the remote assistant for self-adjustments can be considered an alternative to follow-ups, especially in patients with previous hearing experience with CI. Patients who can benefit from the RAF for adjustments to their SP should still be monitored by the clinic, to avoid a drop in CI performance. In our team, patients have a communication channel via email with the audiologists, where they can answer questions and report any problems. It is also possible to establish a routine of annual contact, to check if there are any complaints or difficulties in speech recognition.

There are limitations in this study. The validation of the maps was only based on subjective preference. Furthermore, this study is part of an ongoing project with a larger sample and speech recognition. We believe that this study can be improved not only by emphasizing the benefits and feasibility of the RAF, but also by recognizing its limitations and the characteristics of the suitable patients.

As mentioned earlier, the RAF can be used to create a new map based on the NRT or modify the map already in use, created by an audiologist based on behavioral measures. We must be cautious when offering the possibility of creating new maps based on NRT, so we consider it advisable to use the RAF for patients who have:

- All electrodes active and with adequate impedances and complete insertion of the electrode array.
- Stable maps and periodic returns without complaints.
- Etiology that does not involve morphological changes in the cochlea, such as trauma, otosclerosis, cochlear malformation or meningitis.
- No motor or cognitive disabilities that prevent the remote assistant's usage.
- Presence of intraoperative neural response in at least one electrode, as an indicator of possible postoperatory NRT response.

In cases where the RAF can be offered to patients, it is important to consider the software's limitations. For the cases in which the current levels are close to compliance, in which there is high battery consumption, or when the map in use presents different parameters from the established default (900Hz, 8 max, 25 µs), the software might indicate that the map is not compatible with Master Volume, Bass, and Treble settings, the RAF cannot be used for adjustments, and only the audiologist will be able to adjust the map in use by the patient.

This study is still in progress, evaluating a greater number of adults with CI with the aim of determining the impact of self-adjustment on their speech recognition and daily life.

## Conclusion

We can conclude that SP self-programming in adults with the remote assistant is feasible, since there was no statistically significant difference between the levels of stimulation generated by remote control and those in the behaviorally programmed map. We consider the use of RAF advisable for patients who have: all electrodes active, with adequate impedances and complete insertion of the electrode array; stable maps and periodic returns without complaints; hearing loss etiology that does not involve morphological changes to the cochlea, such as trauma, otosclerosis, meningitis, or cochlear malformation; no motor or cognitive disabilities that make it impossible to manipulate the remote assistant; and presence of intraoperative neural response in at least one electrode.
